# Target Trial Emulation to Improve Causal Inference from Observational Data: What, Why, and How?

**DOI:** 10.1681/ASN.0000000000000152

**Published:** 2023-05-03

**Authors:** Edouard L. Fu

**Affiliations:** Division of Pharmacoepidemiology and Pharmacoeconomics, Department of Medicine, Brigham and Women's Hospital and Harvard Medical School, Boston, Massachusetts

**Keywords:** epidemiology and outcomes, causal inference, observational studies, comparative effectiveness research, target trial

## Abstract

Target trial emulation has drastically improved the quality of observational studies investigating the effects of interventions. Its ability to prevent avoidable biases that have plagued many observational analyses has contributed to its recent popularity. This review explains what target trial emulation is, why it should be the standard approach for causal observational studies that investigate interventions, and how to do a target trial emulation analysis. We discuss the merits of target trial emulation compared with often used, but biased analyses, as well as potential caveats, and provide clinicians and researchers with the tools to better interpret results from observational studies investigating the effects of interventions.

**Glossary of Terms**

- *Confounding bias*: Arises when there is a common cause of the exposure and outcome, leading to bias of the estimated effect. Is not directly solved by target trial emulation and requires measuring and appropriately adjusting for all confounders.
- *Depletion of susceptibles bias*: Bias that can be avoided by target trial emulation. Arises when start of follow-up occurs after treatment assignment. A form of collider stratification bias/selection bias.
- *Immortal time bias*: Bias that can be avoided by target trial emulation. Arises when start of follow-up occurs before treatment assignment such that participants assigned to the treatment group have a period of follow-up during which they cannot experience the outcome and are thus “immortal.”
- *Target trial*: The randomized trial that would answer the causal question of interest.
- *Target trial protocol*: A protocol detailing target trial eligibility criteria, treatment strategies, treatment assignment, outcomes, follow-up, causal estimand, and statistical analysis.
- *Target trial emulation*: Emulation of the specified target trial protocol with observational data.

## Introduction

Routinely collected health care data from claims databases, registries, and electronic health records are increasingly used to answer causal questions on the benefits and harms of medical treatments. Observational studies that are of sufficiently high quality can supplement findings from randomized trials. For example, observational studies can evaluate populations that were underrepresented in clinical trials, perform head-to-head comparisons of interventions (rather than comparisons against placebo), and investigate additional outcomes of interest.^1^ Unfortunately, many observational studies use flawed designs and analyses that introduce avoidable biases, such as immortal time bias.^2,3^ Although many practitioners worry about confounding in observational studies, the effect of these “self-inflicted” biases is often much more severe.^4,5^

Target trial emulation has recently been introduced as a systematic approach to design and analyze observational studies.^6,7^ In the past years, the use of target trial emulation has increased rapidly in the medical literature and also in nephrology research.^8–15^ Nevertheless, its concept and advantages may still be unfamiliar and somewhat mysterious to many readers. Furthermore, not every observational study claiming to use “target trial emulation” is applying the framework correctly.^16^ The aim of this article was therefore to explain what target trial emulation is, why it is needed to improve the quality of observational studies, and how to perform a target trial emulation analysis. An understanding of the target trial emulation framework helps readers to critically appraise published observational studies. In addition, we hope that this article will encourage researchers to adopt the framework when conducting observational studies that aim to answer causal questions on the benefits and harms of medical treatments.

## What is Target Trial Emulation?

Target trial emulation is a framework for designing and analyzing observational studies that aim to estimate the causal effect of interventions.^6,7^ For each causal question on an intervention, one can imagine the randomized trial (the “*target trial*”) that could have been conducted to answer that question. This target trial should be explicitly specified in a *target trial protocol*, which is similar to a trial protocol and includes the following components: eligibility criteria, treatment strategies, treatment assignment, start and end of follow-up, outcomes, causal contrast, and statistical analysis plan. An example of a target trial protocol is presented in Table 1, where the goal was to study the causal effect of renin-angiotensin system inhibitors (RASi) versus calcium channel blockers (CCBs) on outcomes in patients with advanced CKD.^17^ After specifying the target trial protocol, the goal of the investigator is to set up a well-designed observational study that properly emulates each component of the protocol.

**Table 1:** Example of a target trial protocol for an observational study aiming to estimate the causal effect of renin-angiotensin system inhibitors versus calcium channel blockers on outcomes in patients with advanced CKD

Protocol Element	Description	Target Trial	Emulation with Observational Data from the Swedish Renal Registry
Eligibility criteria	Who will be included in this study?	Individuals 18 yr or older under nephrologist care with CKD G4 (*i.e.*, eGFR <30 ml/min per 1.73 m^2^), no history of kidney transplantation, and no use of RASi or CCB in previous 180 d between January 2007 and December 2016	Same as target trial
Treatment strategies	Which precise treatment strategies or interventions will eligible individuals receive?	1. Initiate RASi (ACEi or ARB) only 2. Initiate CCB only	Same as target trial
Treatment assignment	How will eligible individuals be assigned to the treatment strategies?	Randomization, no blinding	Eligible individuals are assigned at baseline to the treatment strategy that their data are consistent with. To emulate randomization, we adjust for the following baseline confounders: age, sex, eGFR, systolic and diastolic blood pressure, medical history (heart failure, arrhythmia, peripheral vascular disease, cerebrovascular disease, ischemic heart disease, diabetes mellitus, hyperkalemia, AKI), medication use (*β*-blocker, thiazide diuretic, potassium-sparing diuretic, statin), and health care use (the total number of hospitalizations in previous year)
Outcomes	What outcomes will be measured during follow-up?	1. Kidney replacement therapy (dialysis or kidney transplantation) 2. All-cause mortality 3. Major adverse cardiovascular events (composite of cardiovascular death, nonfatal myocardial infarction, nonfatal stroke)	Same as target trial. Kidney replacement therapy is registered in the Swedish renal registry; all-cause/cardiovascular mortality is identified from the Swedish death registry; hospitalizations for myocardial infarction or stroke are identified through *ICD-10* codes in the national patient registry
Causal estimand	Which causal estimand will be estimated with the observational data?	Intention-to-treat effect (effect of being randomized to treatment) Per protocol effect (effect of receiving treatment strategy as specified in protocol)	Per protocol effect (effect of receiving treatment strategy as specified in protocol)
Start and end of follow-up	When does follow-up start and when does it end?	Starts at randomization and ends at occurrence of end point, administrative censoring or 5 yr of follow-up	Starts at medication initiation (filled prescription) and ends at occurrence of end point, administrative censoring or 5 yr of follow-up
Statistical analysis	Which statistical analyses will be used to estimate the causal estimand?	Intention-to-treat analysis, non-naïve per protocol analysis	Per protocol analysis: Hazard ratios are estimated using Cox regression while adjusting for baseline confounders with inverse probability of treatment weighting. Weighted cumulative incidence curves are estimated using the Aalen–Johansen estimator^a^

RASi, renin-angiotensin system inhibitor; CCB, calcium channel blocker; ACEi, angiotensin-converting enzyme inhibitor; ARB, angiotensin receptor blocker; G, KDIGO G category.

a A more elaborate description of the statistical analysis can be found in the corresponding paper.

**Figure 1:**
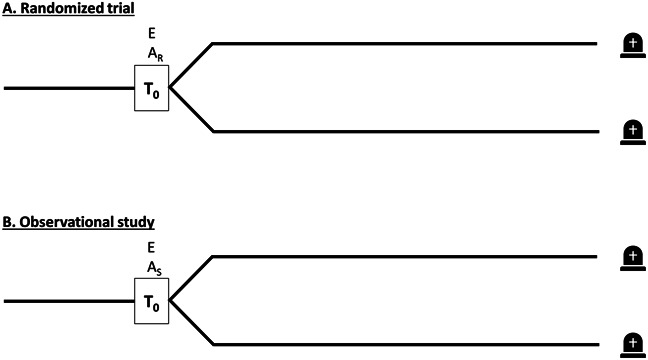
**Alignment of meeting eligibility criteria, treatment assignment and start of follow-up**. (A) the randomized trial (target trial) and (B) the observational study emulating the randomized trial. A_S_, treatment assignment selected by investigator (on the basis of whether data are consistent with being assigned to that treatment strategy); A_R_, randomized treatment assignment; E, meeting all eligibility criteria; T_0_, time zero (=start of follow-up).

To ensure that the target trial protocol properly emulates the design of a randomized trial, it is a key to align the following three components at time zero (often also referred to as baseline) in the observational study (Figure 1).

1. Eligibility criteria are met, that is, all included patients meet the specified inclusion and exclusion criteria.
2. Treatment strategies are assigned.
3. Follow-up is started, that is, we start counting outcomes.

Note that these three components are naturally aligned in randomized trials at the moment of randomization.

It is important to recognize that target trial emulation is a framework for designing and analyzing observational studies. Trial emulation is therefore not linked to only one particular observational study design or statistical analysis: Different target trial protocols may need different observational designs and analyses. Furthermore, the target trial framework can be applied to every causal question on interventions, including surgeries,^18^ vaccinations,^19^ medications,^20^ and lifestyle (diet and physical activity).^21^ Recently, target trial emulation has even been applied to study the causal effects of social interventions^22^ and of changing surgeons' and hospitals' operative volumes.^23,24^ Mendelian randomization studies have also been examined in the target trial framework.^25^ Note that the framework has focused on emulating parallel group trials.

## Why do We Need Target Trial Emulation?

### Observational Studies That Do Not Emulate the Design of a Randomized Trial are Biased

Many published observational studies use flawed study designs that do not properly align treatment assignment and follow-up at time zero and thus do not emulate the design of a randomized trial.^2^ These designs introduce avoidable biases on top of confounding, such as immortal time bias, lead time bias, or selection bias. A recent review found that 57% of included observational studies suffered from immortal time bias and 44% from depletion of susceptibles/prevalent user selection bias.^26^ An intuitive explanation of how these biases arise is in the Supplemental Material.

Many studies reported in the literature that explicitly emulated a target trial obtained results similar to those of randomized trials, whereas observational studies that used biased analyses did not. In the nephrology literature, this has been the case for studies on the timing of dialysis in patients with advanced CKD. The randomized IDEAL trial showed no difference between early versus late dialysis start.^27^ However, virtually all previously published observational studies investigating this causal question showed a strong survival advantage for late dialysis start.^28^ It was recently shown that the discrepancy between these observational studies and the randomized IDEAL trial could be attributed to study design errors—in this case starting follow-up before or after treatment assignment—which introduced immortal time, lead time, and depletion of susceptibles bias^8^ (Table 2). Meanwhile, using target trial emulation led to similar results as the IDEAL trial.^8^ This highlights that the effect of biases from design flaws can be much larger than that of residual confounding.

**Table 2:** Findings of studies investigating timing of dialysis initiation; target trial emulation finds results similar to the randomized trial, whereas previous analyses were biased in either of two ways

Specific Analysis	Correct Study Design	Biases Introduced	Confounding Adjustment Necessary	Hazard Ratio (95% CI) for Early Versus Late Dialysis Initiation
Randomized IDEAL trial	Yes	—	No	1.04 (0.83 to 1.30)
Target trial emulation analysis	Yes	—	Yes	0.96 (0.94 to 0.99)^a^
Common but biased analysis 1	No	Selection bias (depletion of susceptibles), lead time bias	Yes	1.58 (1.19 to 1.78)^b^
Common but biased analysis 2	No	Immortal time bias	Yes	1.46 (1.19 to 1.78)^b^

Based on the study by Fu *et al.*^8^ CI, confidence interval.

a Note that point estimates between IDEAL and target trial emulation analysis are virtually identical (1.04 versus 0.96).

b Hazard ratios obtained with traditional analyses are similar in magnitude to findings from previous observational studies that used the same biased study designs.

The target trial framework also explains the “obesity paradox.” That is, dialysis patients with obesity seem to have better survival than patients with normal body mass index (BMI),^29^ whereas in the general population, high BMI is associated with worse survival. An explanation for the obesity paradox can be depletion of susceptibles bias. Again, this bias occurs because the start of follow-up (at dialysis initiation) is not aligned with the start of the exposure (the occurrence of obesity). Because obesity increases the risk of mortality, individuals with obesity who survive until dialysis must have fewer other risk factors for death than individuals with normal BMI, for whom less selection has taken place.^30^ Target trial emulation not only unravels the paradox but also shows the solution to properly analyze the question: To avoid bias, start of follow-up and exposure should be aligned, and as highlighted in the following section, the investigator should consider specific interventions that lower BMI to make the results interpretable.^31^

### Target trial Emulation Helps to Ask Causal Questions Relevant in Decision-Making

The target trial emulation framework forces investigators to ask causal questions about interventions, leading to findings that are directly useful in decision-making.^32^ What happens when we do not ask questions about interventions? For instance, many observational studies have investigated the causal effect of BMI on outcomes. BMI is not an intervention; patients cannot be randomized to have a certain BMI—a certain BMI can only be achieved through a particular intervention, such as diet, physical exercise, bariatric surgery, or medications (*e.g.*, semaglutide or tirzepatide). These observational studies thus lose the vital information on how a patient attained a different BMI level. Each of the interventions may lower BMI by the same amount but may have completely different causal effects on the outcome. Therefore, the association between BMI and outcomes becomes an amalgamation of each of these interventions, which makes the association difficult to interpret.^33^ The same reasoning applies to other biomarkers, such as blood pressure (antihypertensive agents), LDL cholesterol (statins and ezetimibe), and serum phosphate levels (phosphate binders).

The fact that the causal effect of biomarkers cannot be directly studied does not necessarily mean that the target trial emulation is restrictive—the investigator just needs to reformulate the question in terms of an intervention, just as has been performed to research biomarker targets in real randomized trials.^34–37^ For instance, randomized trials have examined the effects of targeting a certain hemoglobin level through erythropoietin use in patients with anemia and CKD.^34^ This can be emulated in an observational target trial emulation analysis.^15,38^ Phrasing causal questions on biomarkers in terms of interventions also has the large benefit of giving interpretable results that are useful for decision-making, since we now precisely specify how the increase in hemoglobin level is achieved. However, this approach works less well when interventions that modify a biomarker are lacking. In that case, the only option in the target trial emulation framework is to “set” biomarker values to a certain level, for example, comparing treatment strategies “change biomarker level to X mg/dl and keep at this level during follow-up” versus “change biomarker level to Y mg/dl and keep at this level during follow-up.” The investigator should be careful to interpret the results causally because the design suffers from the same amalgamation problem highlighted above. Furthermore, unmeasured time-varying confounding may be an unsurmountable problem because we often do not have a full understanding of all biological processes that influence the biomarker level.

### Target Trial Emulation Helps to Identify Which Confounders to Adjust for

Suppose that an investigator is interested in estimating the causal effect of living donor kidney transplantation versus deceased donor kidney transplantation on graft and recipient survival.^39^ Which confounders should the investigator adjust for: donor characteristics, recipient characteristics, or both? When donor and recipient characteristics are imbalanced, the investigator may be inclined to adjust for both in the observational analysis. Fortunately, thinking about the target trial provides the solution.

In a randomized trial, the investigator randomizes recipients to a kidney transplant from a living donor or a deceased donor. Consequently, the recipients in both groups have similar characteristics. However, living donors will not have characteristics similar to deceased donors in this randomized trial. The potential lower quality of kidneys from deceased donors is part of the treatment. The observational analysis should therefore only adjust for recipient characteristics to emulate this randomization and not for donor characteristics.

### Specification of the Target Trial Dictates the Required Data and Guides the Statistical Analysis

Slightly different research questions may require very different statistical analyses and data. For instance, one investigator may be interested in estimating the causal effect of the treatment strategies “initiate sodium–glucose cotransporter-2 inhibitor” versus “initiate metformin” as first-line treatment in patients with type 2 diabetes, whereas another investigator is interested in the slightly different question “initiate sodium–glucose cotransporter-2 inhibitor and always use during the follow-up period” versus “initiate metformin and always use during the follow-up period*.*” The first researcher needs to measure confounders at baseline only, whereas the second researcher would also require data on time-varying confounders to appropriately answer the question with observational data. Similarly, the first investigator only needs to adjust for baseline confounding in the statistical analysis, whereas the second investigator also needs to appropriately adjust for time-varying confounders, for example, by using inverse probability weighting of marginal structural models.^40,41^ In this way, the required data and statistical analysis logically flow from the specifications in the research question.

However, nuances in the research question rarely become clear in published articles because investigators often do not specifically state their treatment strategies. The target trial protocol forces the investigator to be specific, providing clarity to both investigator and reader.

## How to do Target Trial Emulation

A key component to successfully emulate a target trial is aligning eligibility criteria, assignment of treatment strategies, and start of follow-up at time zero. For each observational study, we can choose from three designs that appropriately emulate a trial design: the active comparator new user design,^42,43^ the clone censor weight design,^44–50^ and the sequential trial design.^10, 51–53^ The type of question determines the design that is required, with some of the most common applications as follows: the active comparator new user design is used to compare the effect of initiating two treatments; the sequential trial design is appropriate when one group starts a treatment, but the other one does not (*e.g.*, starting versus not starting RASi); and the clone censor weight design is useful for grace periods (*e.g.*, patients start a treatment within 6 months of randomization or some event), treatment duration (*e.g.*, take a treatment for 6 versus 12 months), or when treatment is started based on a biomarker level (*e.g.*, start dialysis when eGFR is below 10 ml/min per 1.73 m^2^). The technical details of these designs are outside the scope of this article but are explained in detail elsewhere.^54–60^

In the following sections, we address important considerations when emulating each component of a target trial protocol. For this, we use the example from Table 1, leveraging an active comparator new user design.^17^

### Eligibility Criteria

The eligibility criteria determine who will be included in this study. In the example from Table 1, all eligibility criteria could be emulated: The investigators had access to data on drug dispensing, laboratory findings, and previous kidney replacement therapy. Furthermore, all individuals included in the Swedish Renal Registry were under nephrologist care. Note that a common pitfall is to select eligible individuals based on postbaseline information collected during follow-up. That is, the investigator may be tempted to include individuals who follow a particular treatment strategy during follow-up or who develop the outcome of interest. However, this is incorrect because eligibility criteria determine who is enrolled in the trial. Information collected during follow-up can therefore never be used to determine eligibility.

Sometimes data sources are not sufficient to exactly emulate particular eligibility criteria as specified in the target trial protocol. For instance, administrative claims databases often only contain data on whether tests were performed, but not the results of those tests. Therefore, eligibility criteria such as “serum potassium <5.0 mmol/L” cannot be emulated in many observational data sources. Often, a compromise is necessary between the ideal target trial one would like to emulate and the target trial one can emulate with the available data sources.

### Treatment Strategies

In the example from Table 1, the treatment strategies of interest are “initiate RASi only” versus “initiate CCB only.” This is a typical active comparator new user design, in which interest is in the head-to-head effectiveness of two drugs.^54^ Note that in practice, clinicians are often more interested in the effect of sustained treatment strategies to which patients adhere over time, such as “initiate RASi only and always use during follow-up” versus “initiate CCB only and always use during follow-up.” Treatment strategies should capture these nuances. It is important that the specified treatment strategies do not leave any room for ambiguities since they influence how to specify “follow-up” and “statistical analysis” of the trial emulation protocol (such as whether adjustment for time-varying confounding is needed).

### Treatment Assignment

In a randomized trial, individuals are randomly assigned to one of the treatment strategies. In observational studies, we can only use the observed data to assign eligible individuals to the treatment strategy with which their data are consistent. In the example, eligible individuals who fill a prescription for RASi are thus allocated to the RASi arm, and eligible individuals who fill a prescription for CCB are allocated to the CCB arm. Appropriate emulation of randomization requires sufficient adjustment for all baseline confounders, which need to be measured before treatment assignment. The difficulty is to obtain enough data on confounders to remove residual confounding. The analysis should adjust for all factors that influence outcomes as well as whether someone receives treatment, including factors that may be difficult to quantify, such as frailty.

### Outcomes

Often, administrative data sources do not contain information on outcomes, such as symptoms, quality of life, or pain scores, which can then not be emulated. Another consideration is that outcomes are usually not assessed blinded and systematically in observational data, which can lead to bias. For example, one treatment arm (as defined in “treatment strategies”) may be sicker and therefore have more eGFR measurements during follow-up than the comparator arm, also known as surveillance bias or differential measurement error. Outcomes are then more likely to be found in the sicker treatment arm. One can check for this problem by comparing the number of eGFR measurements between both arms during follow-up.^42^ Note that it is not possible to restrict the analysis to patients who have a certain number of eGFR measurements during follow-up. As discussed in the first part of this section, eligibility criteria need to be met at baseline. Thus, postbaseline information, such as the number of eGFR measurements, can never be used as eligibility criteria since this would lead to selection bias. In the example, all outcomes could be emulated with the data sources at hand.

### Causal Estimand/Causal Contrast

Two causal contrasts that are often estimated in randomized trials are the effect of being randomized to a treatment strategy (intention-to-treat effect) and the effect of receiving the treatment strategy as specified in the protocol (per protocol effect). Because observational studies are not randomized, only per protocol effects can be estimated. However, investigators often refer to the effect of initiating treatment as the intention-to-treat effect in observational studies, which is thus different from the equivalently named effect in randomized trials. A more thorough discussion on these two causal contrasts is in the Supplemental Material.

### Start and End of Follow-Up

In the hypothetical randomized trial from Table 1, follow-up starts at randomization and finishes at reaching an end point, administrative censoring, or 5 years of follow-up. Observational data, however, are not randomized. Therefore, in the observational emulation, follow-up starts when (*1*) the patient is eligible and (*2*) the patient's data are congruent with the start of treatment. For the specified strategies “initiate RASi only” versus “initiate CCB only,” the start of follow-up is clear: medication initiation (a filled prescription). Furthermore, in the observational emulation, the end of follow-up is equivalent to that of the specified target trial.

We now describe two modifications for the start and end of follow-up. First, when comparing the strategies “initiate RASi only” versus “do not initiate RASi,” there is no clear point in time when a patient starts “do not initiate RASi.” A solution to this problem is to analyze the question with sequential trials, which cleverly uses the idea that patient's in the “do not initiate RASi” group can be allocated to this strategy at any point in time when they are eligible.^59,60^ Second, when the interest is in comparing the strategies “initiate RASi only and always use during follow-up” versus “initiate CCB only and always use during follow-up,” the investigator would need to additionally censor individuals (stop follow-up) when they discontinue their assigned treatment (*i.e.*, RASi or CCB) after baseline.

### Statistical Analysis Plan

The statistical analysis plan is similar to the statistical analysis section in articles and specifies which methods were used to estimate the intention-to-treat or per protocol effect. This includes the methods to adjust for confounding (*e.g.*, propensity scores), how missing data were dealt with, and which methods were used to obtain effect estimates (*e.g.*, Cox regression for hazard ratios and Aalen–Johansen estimator for survival curves). A brief description on per protocol analyses is provided in the Supplemental Material, and detailed explanations can be found elsewhere.^61^

## Is Target Trial Emulation a Magic Bullet?

As highlighted previously, target trial emulation has many advantages (Table 3), such as preventing biases due to erroneous study designs. These benefits justify establishment of target trial emulation as the new standard for conducting observational studies on interventions. However, it is not a magic bullet: Such studies may nevertheless have unmeasured or residual confounding. To obtain causal conclusions, it requires measuring all confounders and appropriately adjusting for them. It is important to realize that discussion of whether there is residual confounding and, if so and perhaps more importantly, its magnitude is nuanced, with many influences.^1,62^ These include the particular study question and the design and data sources used. For instance, there is in general less confounding for unintended, harmful effects of drugs (*e.g.*, sodium–glucose cotransporter-2 inhibitors and the risk of diabetic ketoacidosis) than for intended beneficial effects (*e.g.*, sodium–glucose cotransporter-2 inhibitors and the risk of heart failure).^63^ In addition, the degree of confounding for sodium–glucose cotransporter-2 inhibitors and the risk of heart failure would have been smaller when using data from before publication of the large randomized controlled trials that showed large beneficial effects. Comparing a drug (or intervention) with an active comparator can result in less confounding than if the drug was compared with nonusers, although not every comparator is equally suitable.^54^ Some data sources may be more suitable to investigate the effects of treatments than others because they include more detailed confounder information, such as laboratory data or test results.^64^

**Table 3:** Key points of target trial emulation

1. For all observational studies that aim to estimate the causal effect of a treatment, one can imagine the randomized trial that could have been conducted instead (=“target trial”).
2. Target trial emulation is a framework for the design and analysis of observational studies and involves precisely specifying the protocol of the target trial and then emulating each component of the protocol with observational data.
3. The design of randomized trials should be explicitly emulated by carefully aligning eligibility criteria, treatment assignment, and start of follow-up; this prevents avoidable biases, such as immortal time bias and depletion of susceptibles bias.
4. Target trial emulation is not a single study design or analysis; different target trial protocols may need different observational emulation designs and analyses.
5. Target trial emulation does not solve the problem of confounding in observational studies; this requires measuring and appropriately adjusting for all confounders.
6. Increased use of target trial emulation has great potential to improve the quality of causal observational studies; such high-quality studies can be used to complement evidence from randomized controlled trial, *e.g.*, by investigating rare side effects or underrepresented populations.

## Increasing Confidence in Causality from Observational Studies

Benchmarking against existing trial results and control outcome analyses can be used to gain more confidence in findings from observational studies. In benchmarking analyses, the investigator first aims to replicate findings from randomized trials before extending the observational analysis to answer a broader question.^8,65^ If findings from the randomized trial can be accurately replicated in the observational data, more confidence is gained that the data sources and analyses were adequate to adjust for confounding. For instance, in the study on RASi versus CCB in advanced CKD (Table 1),^17^ the investigators first performed a benchmark analysis in patients with CKD G3, for whom there is a large body of trial evidence.^66^ The investigators were able to successfully replicate these trial findings and then extended the analysis to CKD G4–5, the population of interest.^17^ Note that oftentimes, the results from trials and observational studies cannot be readily compared. For instance, the distribution of effect modifiers may differ, outcomes may be measured differently, or the trial and observational study may implement different analyses (intention-to-treat versus per protocol).^67^

Negative control outcomes can be used to detect and adjust for residual bias.^68,69^ These are outcomes for which treatment is not expected to have any effect. An association between the treatment of interest and the negative control outcome therefore points to residual bias. For example, when studying the effects of sodium–glucose cotransporter-2 inhibitors with observational data, investigators can leverage the large body of evidence from randomized trials that showed no effect of these drugs on nonfatal stroke.^70^ Both benchmarking and negative outcome controls require strong subject matter knowledge, often derived from previous randomized trial evidence. Conducting observational studies before there is any trial evidence may therefore be more difficult.

**Figure 2:**
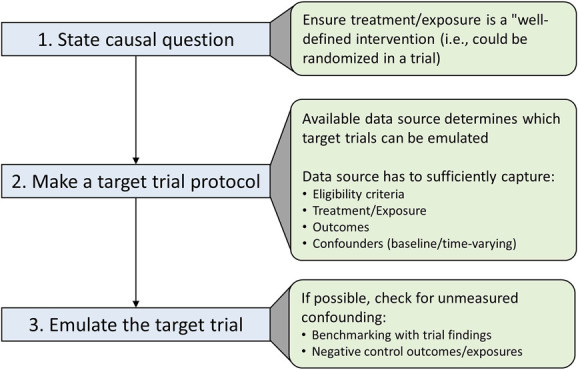
Workflow for conducting observational studies in the target trial emulation framework.

**Figure 3:**
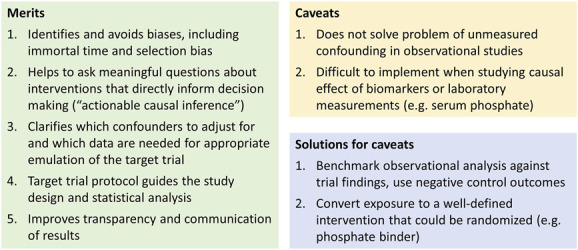
Merits and caveats of the target trial emulation framework.

This article explains target trial emulation, its advantages, and how to do it. Table 3 presents a summary and Figure 2 a workflow for conducting observational studies in this framework. Understanding the principles of target trial emulation helps investigators to critically appraise observational studies and identify avoidable biases. Widespread adoption of the target trial emulation framework as the new standard of observational studies will encourage good practices and improve the quality of observational studies (Figure 3).

## Supplementary Material

## Disclosures

The author has nothing to disclose.

## Funding

E.L. Fu is supported by a Rubicon Grant from the Netherlands Organization for Scientific Research (NWO).

## Acknowledgments

The author thanks Anne Kemmeren for input, critical revisions, and help with the figures and Dr. Friedo Dekker for comments on an earlier version of the manuscript.

## Author Contributions

**Conceptualization:** Edouard L. Fu.

**Formal analysis:** Edouard L. Fu.

**Investigation:** Edouard L. Fu.

**Methodology:** Edouard L. Fu.

**Visualization:** Edouard L. Fu.

**Writing – original draft:** Edouard L. Fu.

**Writing – review & editing:** Edouard L. Fu.

## Supplemental Material

This article contains the following supplemental material online at http://links.lww.com/JSN/E430.

Supplemental Table 1. Specification of the target trial protocol on timing of dialysis and its observational emulation.

Supplemental Figure 1. Alignment of meeting eligibility criteria, treatment assignment and start of follow-up in (A) the randomized trial (target trial) and (B) the observational study emulating the trial.

Supplemental Figure 2. Misalignment of meeting eligibility criteria, treatment assignment and start of follow-up leading to (A) depletion of susceptibles bias/selection bias or (B) immortal time bias in the analysis of a randomized trial.

Supplemental Figure 3. Distinction between confounding and selection bias, illustrated in a randomized trial.

Supplemental Figure 4. Directed acyclic graph of (A) randomized trial and (B) observational study for point interventions.

Supplemental Figure 5. Directed acyclic graph of an observational study where the interest is to estimate per protocol effects of sustained strategies.

## References

[1] Schneeweiss S Patorno E. Conducting real-world evidence studies on the clinical outcomes of diabetes treatments. Endocr Rev. 2021;42(5):658–690. doi:10.1210/endrev/bnab007 33710268 PMC8476933

[2] Hernan MA Sauer BC Hernandez-Diaz S Platt R Shrier I. Specifying a target trial prevents immortal time bias and other self-inflicted injuries in observational analyses. J Clin Epidemiol. 2016;79:70–75. doi:10.1016/j.jclinepi.2016.04.014 27237061 PMC5124536

[3] Suissa S. Immortal time bias in pharmaco-epidemiology. Am J Epidemiol. 2008;167(4):492–499. doi:10.1093/aje/kwm324 18056625

[4] Danaei G Tavakkoli M Hernan MA. Bias in observational studies of prevalent users: lessons for comparative effectiveness research from a meta-analysis of statins. Am J Epidemiol. 2012;175(4):250–262. doi:10.1093/aje/kwr301 22223710 PMC3271813

[5] Dickerman BA Garcia-Albeniz X Logan RW Denaxas S Hernan MA. Avoidable flaws in observational analyses: an application to statins and cancer. Nat Med. 2019;25(10):1601–1606. doi:10.1038/s41591-019-0597-x 31591592 PMC7076561

[6] Hernan MA. Methods of public health research–strengthening causal inference from observational data. N Engl J Med. 2021;385(15):1345–1348. doi:10.1056/nejmp2113319 34596980

[7] Hernan MA Robins JM. Using big data to emulate a target trial when a randomized trial is not available. Am J Epidemiol. 2016;183(8):758–764. doi:10.1093/aje/kwv254 26994063 PMC4832051

[8] Fu EL Evans M Carrero JJ, . Timing of dialysis initiation to reduce mortality and cardiovascular events in advanced chronic kidney disease: nationwide cohort study. BMJ. 2021;375:e066306. doi:10.1136/bmj-2021-066306 34844936 PMC8628190

[9] Fu EL Evans M Clase CM, . Stopping renin-angiotensin system inhibitors in patients with advanced CKD and risk of adverse outcomes: a nationwide study. J Am Soc Nephrol. 2021;32(2):424–435. doi:10.1681/ASN.2020050682 33372009 PMC8054897

[10] Kainz A Kammer M Reindl-Schwaighofer R, . Waiting time for second kidney transplantation and mortality. Clin J Am Soc Nephrol. 2022;17(1):90–97. doi:10.2215/CJN.07620621 34965955 PMC8763155

[11] Shin JI Fine DM Sang Y, . Association of rosuvastatin use with risk of hematuria and proteinuria. J Am Soc Nephrol. 2022;33(9):1767–1777. doi:10.1681/ASN.2022020135 35853713 PMC9529194

[12] Xie Y Bowe B Gibson AK McGill JB Maddukuri G Al-Aly Z. Comparative effectiveness of sodium-glucose cotransporter 2 inhibitors vs sulfonylureas in patients with type 2 diabetes. JAMA Intern Med. 2021;181(8):1043–1053. doi:10.1001/jamainternmed.2021.2488 34180939 PMC8240007

[13] Fotheringham J Latimer N Froissart M, . Survival on four compared with three times per week haemodialysis in high ultrafiltration patients: an observational study. Clin Kidney J. 2021;14(2):665–672. doi:10.1093/ckj/sfaa250 33623692 PMC7886573

[14] Zhang Y Young JG Thamer M Hernán MA. Comparing the effectiveness of dynamic treatment strategies using electronic health records: an application of the parametric g-formula to anemia management strategies. Health Serv Res. 2018;53(3):1900–1918. doi:10.1111/1475-6773.12718 28560811 PMC5980367

[15] Zhang Y Thamer M Kaufman J Cotter D Hernán MA. Comparative effectiveness of two anemia management strategies for complex elderly dialysis patients. Med Care. 2014;52 suppl 2(3):S132–S139. doi:10.1097/mlr.0b013e3182a53ca8 24561752 PMC3933821

[16] Wirth KE Edwards JK Feinstein L Breskin A. When emulating a trial, do as the trialists do: missteps in estimating relative effectiveness of a SARS-CoV-2 vaccine booster dose. Clin Infect Dis. 2022;76(1):176–177. doi:10.1093/cid/ciac700 PMC9452123 36041013

[17] Fu EL Clase CM Evans M, . Comparative effectiveness of renin-angiotensin system inhibitors and calcium channel blockers in individuals with advanced CKD: a nationwide observational cohort study. Am J Kidney Dis. 2021;77(5):719–729.e1. doi:10.1053/j.ajkd.2020.10.006 33246024

[18] Bacic J Liu T Thompson RH, . Emulating target clinical trials of radical nephrectomy with or without lymph node dissection for renal cell carcinoma. Urology. 2020;140:98–106. doi:10.1016/j.urology.2020.01.039 32142726 PMC7255934

[19] Dagan N Barda N Kepten E, . BNT162b2 mRNA covid-19 vaccine in a nationwide mass vaccination setting. N Engl J Med. 2021;384(15):1412–1423. doi:10.1056/nejmoa2101765 33626250 PMC7944975

[20] Xu Y Fu EL Trevisan M, . Stopping renin-angiotensin system inhibitors after hyperkalemia and risk of adverse outcomes. Am Heart J. 2022;243:177–186. doi:10.1016/j.ahj.2021.09.014 34610282

[21] Chiu YH Chavarro JE Dickerman BA, . Estimating the effect of nutritional interventions using observational data: the American Heart Association's 2020 Dietary Goals and mortality. Am J Clin Nutr. 2021;114(2):690–703. doi:10.1093/ajcn/nqab100 34041538 PMC8326054

[22] Rojas-Saunero LP Labrecque JA Swanson SA. Invited commentary: conducting and emulating trials to study effects of social interventions. Am J Epidemiol. 2022;191(8):1453–1456. doi:10.1093/aje/kwac066 35445692 PMC9347019

[23] Madenci AL Wanis KN Cooper Z, . Comparison of mortality risk with different surgeon and hospital operative volumes among individuals undergoing pancreatectomy by emulating target trials in US medicare beneficiaries. JAMA Netw Open. 2022;5(3):e221766. doi:10.1001/jamanetworkopen.2022.1766 35267034 PMC8914572

[24] Madenci AL Wanis KN Cooper Z, . Strengthening health services research using target trial emulation: an application to volume-outcomes studies. Am J Epidemiol. 2021;190(11):2453–2460. doi:10.1093/aje/kwab170 34089045 PMC8799904

[25] Swanson SA Tiemeier H Ikram MA Hernan MA. Nature as a trialist?: deconstructing the analogy between mendelian randomization and randomized trials. Epidemiology. 2017;28(5):653–659. doi:10.1097/ede.0000000000000699 28590373 PMC5552969

[26] Bykov K Patorno E D'Andrea E, . Prevalence of avoidable and bias-inflicting methodological pitfalls in real-world studies of medication safety and effectiveness. Clin Pharmacol Ther. 2022;111(1):209–217. doi:10.1002/cpt.2364 34260087 PMC8678198

[27] Cooper BA Branley P Bulfone L, . A randomized, controlled trial of early versus late initiation of dialysis. N Engl J Med. 2010;363(7):609–619. doi:10.1056/nejmoa1000552 20581422

[28] Susantitaphong P Altamimi S Ashkar M, . GFR at initiation of dialysis and mortality in CKD: a meta-analysis. Am J Kidney Dis. 2012;59(6):829–840. doi:10.1053/j.ajkd.2012.01.015 22465328 PMC3395227

[29] Johansen KL Young B Kaysen GA Chertow GM. Association of body size with outcomes among patients beginning dialysis. Am J Clin Nutr. 2004;80(2):324–332. doi:10.1093/ajcn/80.2.324 15277152

[30] Lajous M Banack HR Kaufman JS Hernán MA. Should patients with chronic disease be told to gain weight? The obesity paradox and selection bias. Am J Med. 2015;128(4):334–336. doi:10.1016/j.amjmed.2014.10.043 25460531 PMC4495879

[31] MacDonald CJ Frenoy P. Re. Weight change and the onset of cardiovascular diseases: emulating trials using electronic health records. Epidemiology. 2022;33(1):e3–e4. doi:10.1097/ede.0000000000001437 34799479

[32] Didelez V. Commentary: should the analysis of observational data always be preceded by specifying a target experimental trial? Int J Epidemiol. 2016;45(6):2049–2051. doi:10.1093/ije/dyw032 27063602

[33] Hernán MA Taubman SL. Does obesity shorten life? The importance of well-defined interventions to answer causal questions. Int J Obes (Lond). 2008;32(S3):S8–S14. doi:10.1038/ijo.2008.82 18695657

[34] Group AC Patel A MacMahon S, . Intensive blood glucose control and vascular outcomes in patients with type 2 diabetes. N Engl J Med. 2008;358(24):2560–2572. doi:10.1056/nejmoa0802987 18539916

[35] Edmonston DL Isakova T Dember LM, . Design and rationale of HiLo: a pragmatic, randomized trial of phosphate management for patients receiving maintenance hemodialysis. Am J Kidney Dis. 2021;77(6):920–930.e1. doi:10.1053/j.ajkd.2020.10.008 33279558 PMC9933919

[36] Singh AK Szczech L Tang KL, . Correction of anemia with epoetin alfa in chronic kidney disease. N Engl J Med. 2006;355(20):2085–2098. doi:10.1056/nejmoa065485 17108343

[37] Drueke TB Locatelli F Clyne N, . Normalization of hemoglobin level in patients with chronic kidney disease and anemia. N Engl J Med. 2006;355(20):2071–2084. doi:10.1056/nejmoa062276 17108342

[38] Thamer M Zhang Y Kaufman J Cotter D Hernan MA. Similar outcomes for two anemia treatment strategies among elderly hemodialysis patients with diabetes. J Endocrinol Diabetes. 2014;1(2). doi:10.15226/2374-6890/1/2/00111 PMC4378694 25834841

[39] Wanis KN Madenci AL Dokus MK, . The meaning of confounding adjustment in the presence of multiple versions of treatment: an application to organ transplantation. Eur J Epidemiol. 2019;34(3):225–233. doi:10.1007/s10654-019-00484-8 30673924

[40] Williamson T Ravani P. Marginal structural models in clinical research: when and how to use them? Nephrol Dial Transplant. 2017;32(suppl 2):ii84–ii90. doi:10.1093/ndt/gfw341 28201767

[41] Robins JM Hernan MA Brumback B. Marginal structural models and causal inference in epidemiology. Epidemiology. 2000;11(5):550–560. doi:10.1097/00001648-200009000-00011 10955408

[42] Xu Y Fu EL Clase CM Mazhar F Jardine MJ Carrero JJ. GLP-1 receptor agonist versus DPP-4 inhibitor and kidney and cardiovascular outcomes in clinical practice in type-2 diabetes. Kidney Int. 2022;101(2):360–368. doi:10.1016/j.kint.2021.10.033 34826514

[43] Desai RJ Patorno E Vaduganathan M, . Effectiveness of angiotensin-neprilysin inhibitor treatment versus renin-angiotensin system blockade in older adults with heart failure in clinical care. Heart. 2021;107(17):1407–1416. doi:10.1136/heartjnl-2021-319405 34088766

[44] Trevisan M Fu EL Xu Y, . Stopping mineralocorticoid receptor antagonists after hyperkalaemia: trial emulation in data from routine care. Eur J Heart Fail. 2021;23(10):1698–1707. doi:10.1002/ejhf.2287 34196082

[45] Petito LC García-Albéniz X Logan RW, . Estimates of overall survival in patients with cancer receiving different treatment regimens: emulating hypothetical target trials in the surveillance, epidemiology, and end results (SEER)-Medicare linked database. JAMA Netw Open. 2020;3(3):e200452. doi:10.1001/jamanetworkopen.2020.0452 32134464 PMC7059023

[46] Wei J Choi HK Neogi T, . Allopurinol initiation and all-cause mortality among patients with gout and concurrent chronic kidney disease: a population-based cohort study. Ann Intern Med. 2022;175(4):461–470. doi:10.7326/m21-2347 35073156 PMC10445508

[47] Lyu H Yoshida K Zhao SS, . Delayed denosumab injections and fracture risk among patients with osteoporosis: a population-based cohort study. Ann Intern Med. 2020;173(7):516–526. doi:10.7326/m20-0882 32716706

[48] Boyne DJ Cheung WY Hilsden RJ, . Association of a shortened duration of adjuvant chemotherapy with overall survival among individuals with stage III colon cancer. JAMA Netw Open. 2021;4(3):e213587. doi:10.1001/jamanetworkopen.2021.3587 33783516 PMC8010592

[49] Caniglia EC Sabin C Robins JM, . When to monitor CD4 cell count and HIV RNA to reduce mortality and AIDS-defining illness in virologically suppressed HIV-positive persons on antiretroviral therapy in high-income countries: a prospective observational study. J Acquir Immune Defic Syndr. 2016;72(2):214–221. doi:10.1097/qai.0000000000000956 26895294 PMC4866894

[50] García-Albéniz X Hernán MA Logan RW Price M Armstrong K Hsu J. Continuation of annual screening mammography and breast cancer mortality in women older than 70 years. Ann Intern Med. 2020;172(6):381–389. doi:10.7326/m18-1199 32092767

[51] Danaei G Garcia Rodriguez LA Fernandez Cantero O Hernan MA. Statins and risk of diabetes: an analysis of electronic medical records to evaluate possible bias due to differential survival. Diabetes Care. 2013;36(5):1236–1240. doi:10.2337/dc12-1756 23248196 PMC3631834

[52] Schmidt M Sorensen HT Pedersen L. Diclofenac use and cardiovascular risks: series of nationwide cohort studies. BMJ. 2018;362:k3426. doi:10.1136/bmj.k3426 30181258 PMC6122252

[53] Wintzell V Svanström H Pasternak B. Selection of comparator group in observational drug safety studies: alternatives to the active comparator new user design. Epidemiology. 2022;33(5):707–714. doi:10.1097/ede.0000000000001521 35944152

[54] Lund JL Richardson DB Sturmer T. The active comparator, new user study design in pharmacoepidemiology: historical foundations and contemporary application. Curr Epidemiol Rep. 2015;2(4):221–228. doi:10.1007/s40471-015-0053-5 26954351 PMC4778958

[55] Johnson ES Bartman BA Briesacher BA, . The incident user design in comparative effectiveness research. Pharmacoepidemiol Drug Saf. 2013;22(1):1–6. doi:10.1002/pds.3334 23023988

[56] Hernán MA Lanoy E Costagliola D Robins JM. Comparison of dynamic treatment regimes via inverse probability weighting. Basic Clin Pharmacol Toxicol. 2006;98(3):237–242. doi:10.1111/j.1742-7843.2006.pto_329.x 16611197

[57] Hernán MA. How to estimate the effect of treatment duration on survival outcomes using observational data. BMJ. 2018;360:k182. doi:10.1136/bmj.k182 29419381 PMC6889975

[58] Cain LE Robins JM Lanoy E Logan R Costagliola D Hernán MA. When to start treatment? A systematic approach to the comparison of dynamic regimes using observational data. Int J Biostat. 2010;6(2):18. doi:10.2202/1557-4679.1212 PMC3406513 21972433

[59] Danaei G Rodriguez LA Cantero OF Logan R Hernan MA. Observational data for comparative effectiveness research: an emulation of randomised trials of statins and primary prevention of coronary heart disease. Stat Methods Med Res. 2013;22(1):70–96. doi:10.1177/0962280211403603 22016461 PMC3613145

[60] Gran JM Roysland K Wolbers M, . A sequential Cox approach for estimating the causal effect of treatment in the presence of time-dependent confounding applied to data from the Swiss HIV Cohort Study. Stat Med. 2010;29(26):2757–2768. doi:10.1002/sim.4048 20803557

[61] Murray EJ Caniglia EC Petito LC. Causal survival analysis: a guide to estimating intention-to-treat and per-protocol effects from randomized clinical trials with non-adherence. Res Methods Med Health Sci. 2021;2(1):39–49. doi:10.1177/2632084320961043

[62] Fu EL van Diepen M Xu Y, . Pharmacoepidemiology for nephrologists (part 2): potential biases and how to overcome them. Clin Kidney J. 2021;14(5):1317–1326. doi:10.1093/ckj/sfaa242 33959262 PMC8087121

[63] Vandenbroucke JP. Observational research, randomised trials, and two views of medical science. PLoS Med. 2008;5(3):e67. doi:10.1371/journal.pmed.0050067 18336067 PMC2265762

[64] Lin KJ Schneeweiss S. Considerations for the analysis of longitudinal electronic health records linked to claims data to study the effectiveness and safety of drugs. Clin Pharmacol Ther. 2016;100(2):147–159. doi:10.1002/cpt.359 26916672

[65] Matthews AA Dahabreh IJ Frobert O, . Benchmarking observational analyses before using them to address questions trials do not answer: an application to coronary thrombus aspiration. Am J Epidemiol. 2022;191(9):1652–1665. doi:10.1093/aje/kwac098 35641151 PMC9437817

[66] Xie X Liu Y Perkovic V, . Renin-angiotensin system inhibitors and kidney and cardiovascular outcomes in patients with CKD: a bayesian network meta-analysis of randomized clinical trials. Am J Kidney Dis. 2016;67(5):728–741. doi:10.1053/j.ajkd.2015.10.011 26597926

[67] Lodi S Phillips A Lundgren J, . Effect estimates in randomized trials and observational studies: comparing apples with apples. Am J Epidemiol. 2019;188(8):1569–1577. doi:10.1093/aje/kwz100 31063192 PMC6670045

[68] Lipsitch M Tchetgen Tchetgen E Cohen T. Negative controls: a tool for detecting confounding and bias in observational studies. Epidemiology. 2010;21(3):383–388. doi:10.1097/ede.0b013e3181d61eeb 20335814 PMC3053408

[69] Shi X Miao W Tchetgen ET. A selective review of negative control methods in epidemiology. Curr Epidemiol Rep. 2020;7(4):190–202. doi:10.1007/s40471-020-00243-4 33996381 PMC8118596

[70] Johansen ME Argyropoulos C. The cardiovascular outcomes, heart failure and kidney disease trials tell that the time to use sodium glucose cotransporter 2 inhibitors is now. Clin Cardiol. 2020;43(12):1376–1387. doi:10.1002/clc.23508 33165977 PMC7724239

