# Supplementary material for: Target Trial Emulation to Improve Causal Inference from Observational Data: What, Why, and How?
Source: J Am Soc Nephrol. 2023 May 3;34(8):1305–14. doi: 10.1681/ASN.0000000000000152 (PMC10400102; doi:10.1681/ASN.0000000000000152)
Supplement: Supplementary file 1 [file jasn-34-1305-s001.docx]

**Supplemental Table of Contents:**

Supplemental Material

Supplemental Table 1. Specification of the target trial protocol on timing of dialysis and its observational emulation.

Supplemental Figure 1. Alignment of meeting eligibility criteria, treatment assignment and start of follow-up in (A) the randomized trial (target trial) and (B) the observational study emulating the trial.

Supplemental Figure 2. Misalignment of meeting eligibility criteria, treatment assignment and start of follow-up leading to (A) depletion of susceptibles bias/selection bias or (B) immortal time bias in the analysis of a randomized trial.

Supplemental Figure 3. Distinction between confounding and selection bias, illustrated in a randomized trial.

Supplemental Figure 4. Directed acyclic graph of (A) randomized trial and (B) observational study for point interventions

Supplemental Figure 5. Directed acyclic graph of an observational study where the interest is to estimate per protocol effects of sustained strategies.

**Supplemental Material:**

*The target trial*

In this section, we provide an intuitive explanation why biases arise when start of follow-up and treatment assignment are not aligned at time zero/baseline, applied to the target trial investigating the effect of early (eGFR of 10-14 ml/min/1.73m^2^) versus late (eGFR 5-7 ml/min/1.73m^2^) dialysis initiation on all-cause death in patients with advanced CKD. The target trial protocol for this example is specified in **Supplemental** **Table 1**. According to the protocol, this randomized trial would include individuals at the moment their eGFR falls between 10-20 ml/min/1.73m^2^ (**Supplemental** **Figure 1**). Individuals who also comply with the other eligibility criteria are then randomly assigned to the treatment strategies “start dialysis with an eGFR between 10-14 ml/min/1.73m^2^” vs. “start dialysis with an eGFR between 5-7 ml/min/1.73m^2^”. Follow-up should also be started at that moment to align the three key elements at time zero. This ensures that deaths that occur after randomization but before the start of dialysis are appropriately counted as outcomes ^1,2^.

*Aligning eligibility, treatment assignment and start of follow-up*

Naturally, observational studies addressing this causal question should adhere to the same design principle as randomized trials, and ensure that eligibility, treatment assignment and follow-up are aligned at time zero (**Supplemental Figure 1**)^1-3^. However, virtually all previously published observational studies investigating the causal question of when to start dialysis either started follow-up before or after treatment assignment (**Supplemental Figure 2**), and therefore nearly all showed a survival advantage for late dialysis start^4^. We recently showed that the discrepancy between these observational studies and the randomized IDEAL trial^5^ (which showed no difference between early vs. late dialysis start) could be attributed to study design errors that introduced immortal time, lead time and depletion of susceptibles bias, rather than unmeasured confounding^2^ (**Table 2**).

*A tale of three investigators*

To understand why these biases arise and how target trial emulation avoids them, imagine that we actually conducted the randomized trial specified in **Supplemental** **Table 1**. To simplify the thought experiment, suppose that our randomized trial had an infinitely large sample size and was perfectly conducted: there were no losses to follow-up and each included individual fully adhered to their assigned strategies (i.e. all individuals that were assigned to the eGFR 5-7 group started dialysis with an eGFR of 5-7 ml/min/1.73m^2^, and all individuals assigned to the eGFR 10-14 group started dialysis with an eGFR of 10-14 ml/min/1.73m^2^). The data from the randomized trial are then analyzed by three investigators. Investigator 1 starts follow-up at the moment of treatment assignment/randomization; she finds a hazard ratio of 1.0 and concludes that there is no causal effect of early vs. late dialysis initiation on mortality. Investigator 2 disagrees and argues that follow-up should start at dialysis initiation, rather than at the moment of randomization (**Supplemental** **Figure 2A**). Only individuals that started dialysis should be included, and only deaths that occurred after dialysis initiation should be counted. Therefore, he removes from the analysis all individuals who died between randomization and dialysis start. He finds a hazard ratio of 1.5 for early vs. late dialysis initiation and concludes that early dialysis initiation is harmful. Investigator 3 thinks both of them are wrong: follow-up should start at randomization, but only individuals that survive long enough to start dialysis should be included in the analysis (**Supplemental** **Figure 2B**). He therefore allocates survivors that started dialysis with an eGFR of 5-7 to the late start group, and survivors who started dialysis with an eGFR of 10-14 to the early start group. He also finds a hazard ratio of 1.5 and concludes that early dialysis initiation is harmful.

*How does bias arise if the trial design is not emulated?*

Of course, we know that investigator 1 is right. However, many observational studies analyzed their data like investigators 2 and 3, leading to substantially biased results^4^. Below, we explain intuitively why the wrong analyses led to biased associations favoring late dialysis initiation.

Investigator 2 started follow-up at dialysis initiation, which leads to “depletion of susceptibles” bias and lead time bias^6^. These two biases work in opposite directions, in the sense that “depletion of susceptibles” artificially favors late dialysis initiation, whereas “lead time” bias favors early dialysis initiation. In most observational studies the “depletion of susceptibles” bias was stronger since the overall result favored late dialysis initiation. However, some of the older observational studies have shown a benefit for early dialysis initiation due to “lead time” bias^7^.

Both biases arise as a result of comparing two groups that are no longer similar. At the moment of randomization, both the early and late start arm have a similar prognosis, and include a similar proportion of high risk individuals (i.e., there is no confounding) (**Supplemental Figure 2A and 3**). However, at the moment of dialysis initiation, the early and late start arm are no longer similar: the individuals that start dialysis with an eGFR of 5-7 had to survive longer than the individuals who started dialysis with an eGFR of 10-14.

In the case of “depletion of susceptibles” bias, the reasoning is that more high-risk individuals will have died in the eGFR 5-7 arm than the 10-14 arm, leaving a group of healthier survivors in the late start arm. The late start arm has been progressively depleted of patients who are most likely to die (i.e., depletion of susceptibles), and will only consist of survivors who likely do not have other risk factors for death. Therefore, comparing these healthy survivors in the late start arm with the sicker population remaining in the early start arm leads to an unfair advantage for the late start arm, hence the hazard ratio of 1.5 for early vs. late dialysis initiation. The depletion of susceptibles bias is an example of collider stratification bias (a form of selection bias): it arises due to selecting survivors into the study, and this is distinct from confounding: the selection bias can also occur in randomized trials (which do not have confounding) by starting follow-up after treatment assignment^8^ (**Supplemental Figure 3**).

On the other hand, in the case of “lead time” bias, the reasoning is that the individuals surviving until the late start group are farther along in their disease course and will have a shorter time left to live. Meanwhile, early starters are earlier in their disease course and have a longer period to live. Comparing these two groups then leads to an artificial benefit for early starters. Note that this is exactly opposite from “depletion of susceptibles” bias, where late starters are healthier than early starters because susceptibles do not survive until dialysis start. For both biases, the crux of the problem is that time zero of follow-up is not correctly emulated.

Investigator 3 started follow-up at randomization but then used future information to classify individuals into exposure groups. This introduces immortal time: patients are required to survive until dialysis start, because those who died before having the opportunity to start dialysis are removed from the analysis^9,10^ (**Supplemental Figure 2B**). Therefore, all patients included in the analysis are immortal from the start of follow-up until the start of dialysis. Because the immortal time is larger in the late initiation arm than the early arm, immortal time *bias* is introduced which favors the late initiation arm^2^. Hence, investigator 3 also finds a hazard ratio of 1.5 for early vs. late dialysis initiation. Note that the distinction between the two biases is when follow-up is started: at the moment of randomization or at the moment of dialysis initiation. Both analyses are biased because assignment of treatment strategies and start of follow-up are not aligned at a the same moment in time for both treatment arms^5^.

**Supplemental Table 1**. Specification of the target trial protocol on timing of dialysis and its observational emulation.

| **Component** | **Target trial** | **Emulation in Swedish Renal Registry** |
| --- | --- | --- |
| Eligibility | Adults (≥18 years) with eGFR between 10-20 ml/min/1.73m^2^ and previous eGFR between 10-30 ml/min/1.73m^2^ (calculated with CKD-EPI 2009 equation), and no history of kidney transplantation or dialysis between January 2007 and December 2016. | Same as target trial. In addition, individuals are required to have a measurement of systolic blood pressure, diastolic blood pressure, calcium, phosphate, albumin and hemoglobin |
| Treatment strategies | “start dialysis with an eGFR between 5-7 ml/min/1.73m^2^” vs. “start dialysis with an eGFR between 10-14 ml/min/1.73m^2^” | Same as target trial. |
| Treatment assignment | Eligible individuals are randomly assigned to one of the strategies and are aware of the treatment strategy they are assigned to (i.e., no blinding). | Randomization is emulated via cloning of individuals and assigning each replicate to a different treatment strategy. Cloning is used since each individual can be allocated to both strategies at baseline |
| Follow-up | For each individual follow-up starts at the time of assignment to a strategy (and all eligibility criteria are met) and ends at the occurrence of death, major cardiovascular event, administrative censoring (June 2017) or 5 years, whichever comes first. | Follow-up starts at the first time when all eligibility criteria are met. |
| Primary end point | 1. All-cause mortality 2. Major adverse cardiovascular events (composite of cardiovascular death, non-fatal myocardial infarction or non-fatal stroke) | Same as target trial. |
| Causal contrast | Intention-to-treat effect (effect of treatment assignment).  Per protocol effect (effect of receiving treatment according to the trial protocol). | Per protocol effect |
| Statistical analysis | Intention-to-treat analysis.  Non-naïve per protocol analysis: Individuals are artificially censored when they deviate from their assigned strategy as follows:   1. If an individual initiates dialysis but his/her eGFR is not in the range of the treatment strategy he/she was assigned to. 2. If an individual does not initiate dialysis but his/her eGFR has already passed below the assigned eGFR value to start dialysis.   Note that inverse probability weighting is required also in a randomized trial to validly estimate the per-protocol effect. IP weights are estimated as a function of the following time-fixed and time-varying confounders:  Time-fixed variables: age, sex, calendar year, baseline eGFR, primary kidney disease  Time-fixed and time-varying variables: eGFR, previous eGFR value, systolic and diastolic blood pressure, total calcium, phosphate, albumin, hemoglobin,  comorbidities (acute coronary syndrome, ischemic heart disease, heart failure, diabetes, valvular heart disease, stroke, other cerebrovascular disease, atrial fibrillation, other arrhythmias, peripheral vascular disease, chronic obstructive pulmonary disease, venous thromboembolism, cancer, liver disease, fracture), medication use (beta blockers, calcium channel blockers, diuretics, renin-angiotensin system inhibitors, lipid-lowering therapy, potassium binder, phosphate binder, erythropoietin stimulating agents, vitamin D, digoxin, nitrates, antiplatelet therapy, anticoagulants, sodium bicarbonate) and hospitalizations (total number of hospitalizations in previous year, cardiovascular hospitalization in previous year). | Same as per protocol analysis. We created an expanded dataset including two replicates for each included individual and assigned one replicate to each treatment strategy. We adjusted for baseline and time-varying confounders and assumed that adjustment for these variables was sufficient to adjust for informative censoring. |

**Supplemental Figure 1**. Alignment of meeting eligibility criteria, treatment assignment and start of follow-up in (A) the randomized trial (target trial) and (B) the observational study emulating the trial.


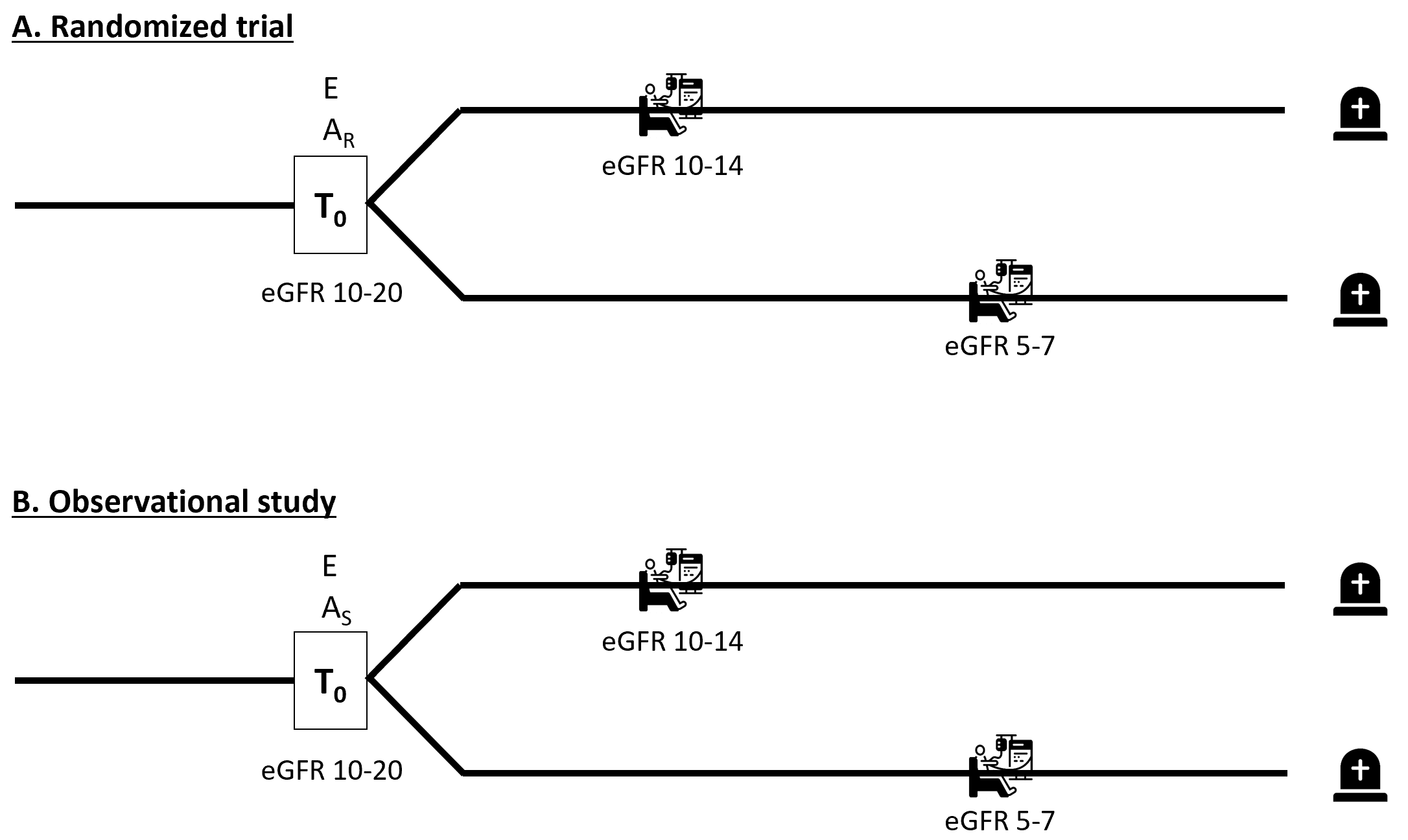


A_S_ = treatment assignment selected by investigator (based on whether data are consistent with being allocated to that treatment strategy); A_R_ = randomized treatment assignment; E = meeting all eligibility criteria; T_0_ = time zero (= start of follow-up).

**Supplemental Figure 2**. Misalignment of meeting eligibility criteria, treatment assignment and start of follow-up leading to (A) depletion of susceptibles bias/selection bias or (B) immortal time bias in the analysis of a randomized trial.


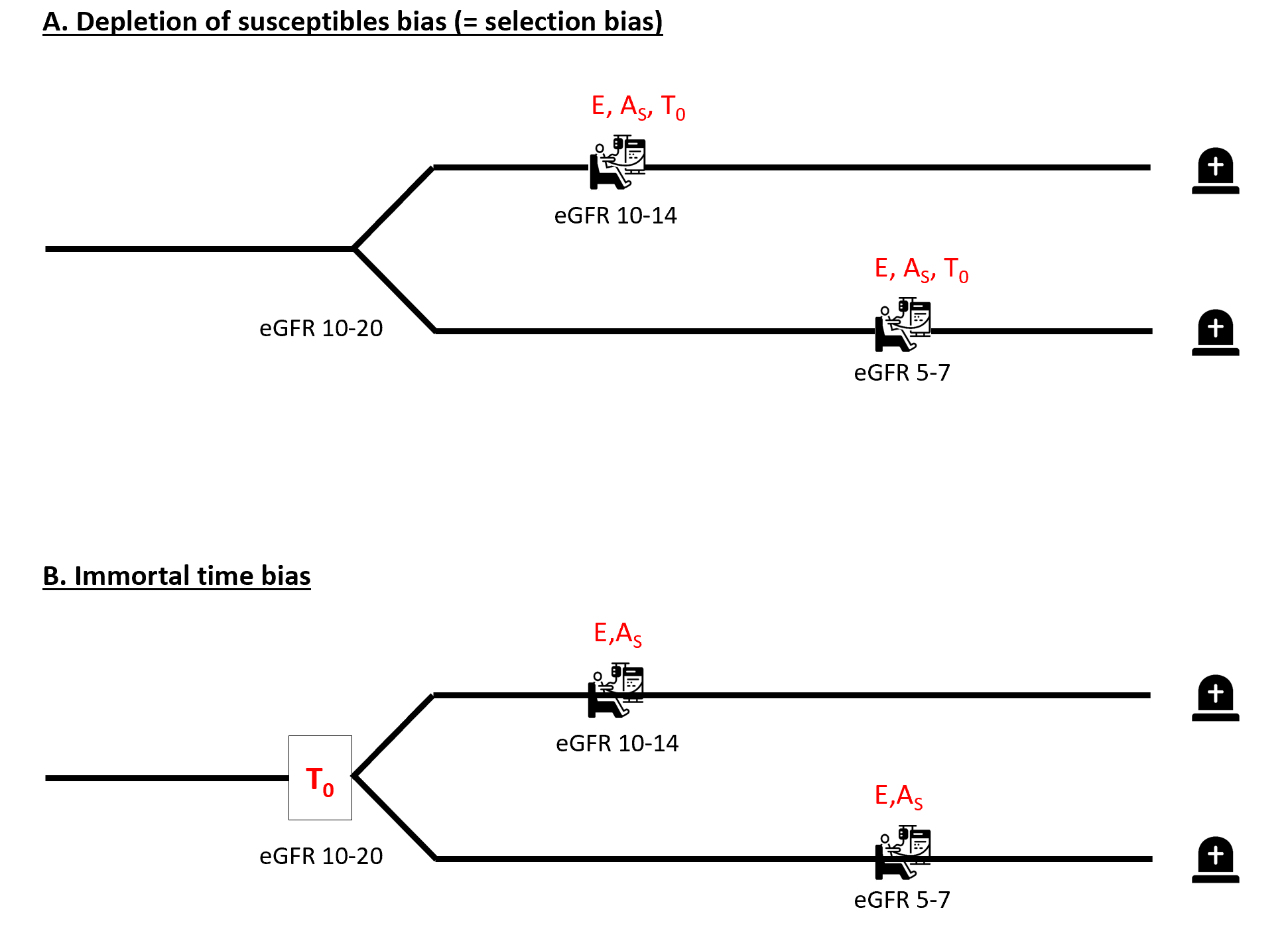


A_S_ = treatment assignment selected by investigator (based on whether data are consistent with being allocated to that treatment strategy); E = meeting all eligibility criteria; T_0_ = time zero (= start of follow-up).

Misalignment of start of follow-up and treatment assignment at a single moment in time for both treatment groups leads to depletion of susceptibles bias (a form of selection bias) or immortal time bias.

**Supplemental Figure 3**. Distinction between confounding and selection bias, illustrated in a randomized trial.


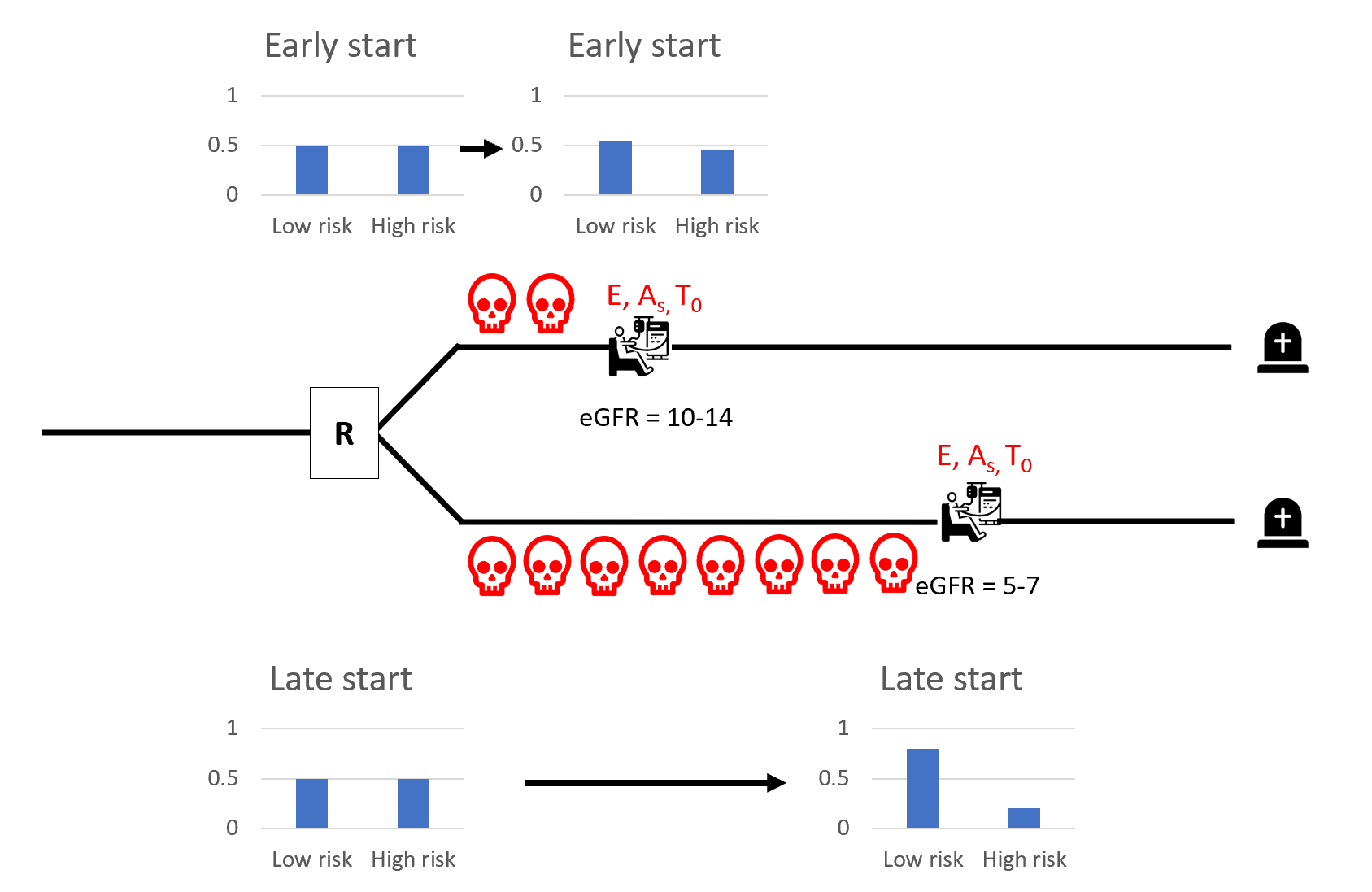


At randomization (denoted by R), the early and late start arms have a similar proportion of high risk patients (in this example 50%). This is expected because of randomization. However, at the start of dialysis the proportion of high risk individuals is no longer similar between both arms, because more selection has taken place in the late start arm (= selection bias): the late start group had to survive longer to be included in the study. This highlights the distinction between confounding and selection bias.

**Point interventions vs. sustained strategies**

Based on their duration, treatments can be characterized as *point interventions* or as *sustained strategies*, which has important implications for the statistical analysis and interpretation of results. As the name implies, point interventions happen at a single point in time. Examples are vaccinations or a single surgery, but also *initiation* of some prolonged treatment (e.g. starting a medication course). On the other hand, sustained strategies are treatments that are given over time, such as drugs that are taken daily. Our interest often lies in estimating the causal effects of sustained strategies, since most treatments are sustained (e.g. drugs are usually given over a longer period in time)^11^. The following sections revisit implications in the analysis for both point and sustained treatment strategies, and link this to intention-to-treat and per protocol analyses.

**Intention-to-treat effects in randomized trials**

In randomized clinical trials, the intention-to-treat (ITT) effect is defined as the effect of being *assigned* to some treatment^12^. In **Supplemental Figure 4** this is denoted by the relationship between Z and Y. An ITT analysis of a randomized trial will not suffer from confounding, because treatment assignment is randomized. Note that the effect of treatment assignment is not necessarily equal to the effect of treatment initiation, which is denoted by the arrow between A and Y in **Supplemental Figure 4**. The ITT effect will only coincide with the effect of treatment initiation if all individuals in the trial start their assigned treatment.

Observational studies do not have randomization and therefore one cannot estimate the effect of treatment assignment. Thus, when investigators use the term “intention-to-treat” for observational studies, they typically refer to the effect of *initially starting* a treatment, which is actually the per protocol effect of a point intervention.

A

Y

L

Observational study

Z

A

Y

L

Randomized trial

**Supplemental Figure 4**. Directed acyclic graph of (A) randomized trial and (B) observational study for point interventions. Z = treatment assignment; A = (starting) treatment; L = confounder; Y = outcome. The intention-to-treat effect is defined as the effect of Z on Y, and can only be estimated in the randomized trial. The per protocol effect of point intervention A is defined as the effect of starting treatment A on outcome Y, and is the only effect that can be estimated in an observational study. Note that valid estimation of the per protocol effect requires adjustment for the confounder L.

**Per protocol effects of point interventions in observational studies**

The per protocol effect is defined as the “effect of receiving treatment according to the trial protocol”. E.g., if the target trial protocol specifies that the aim is to estimate the causal effect of treatment initiation – a point intervention – then this is our per protocol effect. In the observational study the investigator also needs to adjust for all baseline confounders^12^, since individuals who decide to take treatment likely have a different prognosis from those who do not. Estimated association will be biased if there are unmeasured confounders. An advantage of point interventions is that confounding adjustment is only needed at baseline; no adjustment for time-varying confounding is required (in the absence of loss to follow-up).

However, per protocol analyses of point interventions also have a disadvantage. Patients will remain in their initial treatment group, even if they discontinue the treatment or switch to the comparator treatment during follow-up. Thus, the magnitude of nonadherence during follow-up will influence the per protocol effect: if one observational study analyzes data from a healthcare system where adherence is 80%, and another observational study analyzes data from another healthcare system where adherence is only 60%, then both observational studies may find a different per protocol effect (e.g. a hazard ratio of 0.70 in the former, and 0.90 in the latter). Thus, results from such observational studies may be difficult to generalize to other settings with different adherence patterns^12^. Non-adherence is often large in routine clinical practice: in an observational study on the safety of SGLT-2 inhibitors, 60% of patients discontinued their treatment during follow-up^13^. Note that nonadherence is not an issue for true point interventions such as vaccinations (if only one vaccine is needed) or single-time surgery, as these are only given once.

**Per protocol effects of sustained strategies in observational studies**

Instead of specifying a per protocol effect of a point intervention, the investigator could also specify a per protocol effect of a sustained strategy^14^. For instance, an investigator may be interested in estimating the causal effect of “initiating drug A *and always using it during follow-up, unless contraindications develop*” vs. “never initiating drug A during follow-up” (**Supplemental Figure 5**). This is a sustained strategy because treatment is not given once, but over a longer time period. The advantage of per protocol effects of sustained strategies is that adherence patterns will not influence this effect: we are estimating what happens if everyone follows their assigned strategy. However, unbiased estimation of per protocol effects of sustained strategies requires (1) high-quality *longitudinal* data on confounders and treatment adherence to adjust for time-varying confounding due to incomplete adherence, and (2) appropriate methods, i.e. G-methods that can handle time-varying confounding, such as inverse probability weighting (marginal structural models) or the G-formula^14,15^. It is often difficult to capture all reasons why patients continue or discontinue their treatment in administrative databases; if there are any unmeasured time-varying confounders, estimated association will be biased.

A_2_

A_0_

A_1_

L_0_

L_1_

Y

L_2_

**Supplemental Figure 5**. Directed acyclic graph of an observational study where the interest is to estimate per protocol effects of sustained strategies. Note that for simplicity, we only show three timepoints. A_i_ = treatment at timepoint i; L = confounder; Y = outcome. The per protocol effect of sustained strategy A can be defined as always using treatment A (A_0_=1, A_1_=1, A_2_=1) vs. never using treatment A (A_0_=0, A_1_=0, A_2_=0). Note that valid estimation of per protocol effects requires appropriate adjustment for baseline (L_0_) and time-varying confounders (L_1_, L_2_) using G-methods.

To estimate per protocol effects, the investigator should censor patients once they do not adhere anymore to their assigned treatment strategy (i.e. follow-up is stopped). For instance, if an individual stops treatment A during month 3 of follow-up, and this was not due to a contraindication, he or she is censored at that moment. This censoring is nearly always informative, since patients who adhere to the protocol will be different from those who do not. Simply stopping their follow-up would then bias the results. To obtain an unbiased result, adjustment for all time-varying confounders is needed with appropriate methods, such as inverse probability weighting. Intuitively, inverse probability weighting gives each person a weight such that all measured confounders no longer predict adherence to the protocol, removing bias from measured confounders. In the causal diagram, inverse probability weighting removes the arrows form the confounders (L_0_/L_1_/L_2_) into treatment (A_0_/A_1_/A_2_). For example, in the observational study on timing of dialysis, adjustment for time-varying confounding moved the risk difference from -0.13% to -3.61% for the outcome major adverse cardiovascular events^2^.

**Supplemental references**

1. Sjolander A, Nyren O, Bellocco R, Evans M. Comparing different strategies for timing of dialysis initiation through inverse probability weighting. *Am J Epidemiol.* 2011;174(10):1204-1210.

2. Fu EL, Evans M, Carrero JJ, et al. Timing of dialysis initiation to reduce mortality and cardiovascular events in advanced chronic kidney disease: nationwide cohort study. *BMJ.* 2021;375:e066306.

3. Hernan MA, Sauer BC, Hernandez-Diaz S, Platt R, Shrier I. Specifying a target trial prevents immortal time bias and other self-inflicted injuries in observational analyses. *J Clin Epidemiol.* 2016;79:70-75.

4. Susantitaphong P, Altamimi S, Ashkar M, et al. GFR at initiation of dialysis and mortality in CKD: a meta-analysis. *Am J Kidney Dis.* 2012;59(6):829-840.

5. Cooper BA, Branley P, Bulfone L, et al. A randomized, controlled trial of early versus late initiation of dialysis. *N Engl J Med.* 2010;363(7):609-619.

6. Stovitz SD, Banack HR, Kaufman JS. 'Depletion of the susceptibles' taught through a story, a table and basic arithmetic. *BMJ Evid Based Med.* 2018;23(5):199.

7. Korevaar JC, Jansen MA, Dekker FW, et al. When to initiate dialysis: effect of proposed US guidelines on survival. *Lancet.* 2001;358(9287):1046-1050.

8. Hernan MA, Hernandez-Diaz S, Robins JM. A structural approach to selection bias. *Epidemiology.* 2004;15(5):615-625.

9. Suissa S. Immortal time bias in pharmaco-epidemiology. *Am J Epidemiol.* 2008;167(4):492-499.

10. Levesque LE, Hanley JA, Kezouh A, Suissa S. Problem of immortal time bias in cohort studies: example using statins for preventing progression of diabetes. *BMJ.* 2010;340:b5087.

11. Hernan MA, Hernandez-Diaz S, Robins JM. Randomized trials analyzed as observational studies. *Ann Intern Med.* 2013;159(8):560-562.

12. Hernan MA, Robins JM. Per-Protocol Analyses of Pragmatic Trials. *N Engl J Med.* 2017;377(14):1391-1398.

13. Fu EL, D'Andrea E, Wexler DJ, Patorno E, Paik JM. Safety of Sodium-Glucose Cotransporter-2 Inhibitors in Patients with CKD and Type 2 Diabetes: Population-Based US Cohort Study. *Clin J Am Soc Nephrol.* 2023.

14. Murray EJ, Caniglia EC, Petito LC. Causal survival analysis: A guide to estimating intention-to-treat and per-protocol effects from randomized clinical trials with non-adherence. *Research Methods in Medicine & Health Sciences.* 2021;2(1):39-49.

15. Toh S, Hernan MA. Causal inference from longitudinal studies with baseline randomization. *Int J Biostat.* 2008;4(1):Article 22.
